# Scientific Research: What it Means to Me

**DOI:** 10.4103/0973-1229.33003

**Published:** 2008

**Authors:** Jayant V. Narlikar

**Affiliations:** * *Founder Director, Inter-University Centre for Astronomy and Astrophysics (IUCAA) 1989-2003, Pune; President, Cosmology Commission of the International Astronomical Union, 1994-97. Astrophysicist and Science populariser*

**Keywords:** *Serendipity*, *Intuition*, *Isaac Newton*, *Kelvin*, *Maxwell*, *Hoyle*, *Brian Josephson*, *Seeding*, *Growing*, *Eureka*, *Non-Conformism*, *Creativity*, *Cambridge*

## Abstract

This article gives a personal perception of the author, of what scientific research means. Citing examples from the lives of all time greats like Newton, Kelvin and Maxwell he stresses the agonies of thinking up new ideas, the urge for creativity and the pleasure one derives from the process when it is completed. He then narrates instances from his own life that proved inspirational towards his research career. In his early studenthood, his parents and maternal uncle had widened his intellectual horizons while in later life his interaction with Fred Hoyle made him take up research challenges away from the beaten path. He concludes that taking up an anti-Establishment stand in research can create many logistical difficulties, but the rewards of success are all the more pleasing.

## Introduction

At a time when purely commercial attitude prevails in education, it has become necessary to state the obvious: that pure science, motivated as it is by the thirst for knowledge, forms the foundation of the superstructure of science and technology that has become the mainstay of our present civilization. But even more than that it needs to be emphasized that pure science today is a natural extension of the age old and continuing efforts of intellectuals to understand the mysteries of nature. The ancient sages searching for enlightenment went through extended periods of agony, which only made the attainment of goal a matter of great ecstasy. To me scientific research means participation in an intellectually challenging exercise which involves its moments of agony and ecstasy.

Many scientists have experienced similar moments in their search for truth. Agony that you go through when you are searching for the elusive solution to a problem – a solution that, you feel in your bones, must exist. Ecstasy that you experience when you find it.

Indeed, history of science is full of inspiring names of scientists who have gone through such cycles of agony and ecstasy. It may be worth going through a few such examples. When I made my selection for recounting them here I discovered that inadvertently, all of them belong to my *alma mater*, the University of Cambridge! I may not be exaggerating when I say that it is hard to think of any other institution with such a long tradition of scientific discoveries.

## Isaac Newton

Now more than 350 years have passed since the birth of Isaac Newton. The science we enjoy today rests on the pioneering work of Newton done at a time when there was hardly any basic infrastructure. How did he function as a scientist?

Newton was a rare combination of genius, hard work and whimsicality. It is often stated in scientific folklore that he thought of the inverse square law of gravitation when a falling apple hit him on the head, as he was relaxing in his home garden. This may suggest that only because of the serendipitous fall of the apple Newton thought of gravitation and this may also imply a “eureka” type discovery. Nothing can be more unfair to Newton's abilities, to the work of his predecessors like Kepler and Galileo and to the history of astronomy. It would be hard, if not impossible, even for the most sophisticated instruments of today, to deduce the inverse square law by accurately measuring the acceleration of a falling apple. The inverse square law was deduced not from the fall of an apple but by attempts to understand the motions of planets and the moon. Newton had Kepler's laws to explain. He invented a new branch of mathematics to calculate planetary trajectories – a branch now known as *calculus*.

The Nobel Laureate astrophysicist Professor S. Chandrasekhar, has remarked that he had solved the propositions in Newton's *Principia* and found, on almost every occasion, that Newton's original solutions three centuries earlier were more elegant than his own modern attempts! But the final product *Principia* does not reveal the painstaking efforts that went into it. Newton's biographies have given us glimpses into the agonies of scientific discovery that he must have gone through.

The following accounts illustrate how his contemporaries saw Newton:

As when he has been in the hall at dinner he has quite neglected to help himself and the cloth has been taken away before he has eaten anything. That sometimes when on surplice days, he would go toward S. Mary's church, instead of college chapel or perhaps has gone in his surplice to dinner in the hall. That when he had friends to entertain at his chamber, if he stepped in to his study for a bottle of wine and a thought came into his head, he would sit down to paper and forget his friends.

P. Stukeley

[From Never at Rest by Richard Westfall, Cambridge 1980, p. 191]

He always kept close to his studies, very rarely went a visiting and had a few visitors, excepting two or three Persons, Mr. Ellis of Keys, Mr. Lougham [called Laughton in his other letter] of Trinity and Mr. Vigani, a Chemist in whose company he took much Delight and Pleasure at an Evening, when he came to wait upon him. I never knew him take any Recreation or Pastime, either in Riding out to take y^e^ Air, Walking, Bowling or any other Exercise whatever, Thinking all Hours lost, y^t^ was not spent in his studies, to w^ch^ he kept so close, y^t^ he seldom left his Chamber, unless at Term Time, when he read in y^e^ schools, as being Lucasianus Professor. He very rarely went to Dine in y^e^ Hall unless upon some Public Days and then, if he has not been minded, would go very carelessly, w^th^ Shooes down at Heels, Stockings unty'd, surplice on and his head scarcely comb'd.

Humphrey Newon

[From Never at Rest by Richard Westfall, Cambridge 1980, p. 191-192]

This is my first example. Let me now jump across two centuries to look at two other great scientists.

## Kelvin and Maxwell

In his early life Lord Kelvin was known by his family name Thomson. This story refers to Thomson and another young man Parkinson when both were competing for the top rank in their Cambridge Tripos examination. In the end Parkinson topped the list and Thomson was ranked second, with the rest of the pack far behind.

There was one particularly difficult question, which only the two had answered correctly. What struck the examiner most was the similarity of their answers, so much so that he suspected malpractice. Did one boy copy the other's answer? He called Parkinson for an interview –

“Tell me, how did you manage to solve such a difficult question?” he asked Parkinson.

“Sir, I occasionally read research journals. I came across a paper wherein the (anonymous) author had solved this problem.” He gave the reference to the paper.

The examiner who himself had taken the problem from that very same paper was impressed. He complimented the boy for venturing out of the teaching syllabus and reading new articles. Dismissing him with a pat on the back he called Thomson and asked him somewhat aggressively: “I would like to know how you solved this problem. Parkinson who solved it saw the solution in a research paper. Don't tell me that you also saw it there.”

“No, Sir!” replied the future Lord Kelvin. “I wrote that paper.”

And that about sums up the intellectual calibre of Thomson, a.k.a. Kelvin.

James Clerk Maxwell was another aspirant for the top rank in his batch. Indeed so confident was he that he did not bother to attend the Result Declaration ceremony at the Senate House. Instead he sent his valet with the instruction, “Find out who is second.” For, he was curious to know who amongst his rivals would be second.

The valet came back in due course. “Well! Who was second?” asked Maxwell.

“You, Sir!” said the valet!

Maybe, Maxwell was disappointed at not making it to the top of the exams as, probably was Thomson. But both made it to the top of scientific research through outstanding contributions to electricity, magnetism, thermodynamics, etc. In a scientific career what, in the last analysis, matters most is originality. So not all is lost if one failed to secure the top rank in one's examination! I can name many persons who topped the merit list by sheer hard work and rote learning but failed to make the grade in scientific research.

After all, do we know what Parkinson did in later life? What did the student who surpassed Maxwell in the exams achieve later in his life? We do not know!

## Brian Josephson

Coming to the present times, I will now tell the story of a genius who was a fellow student with me at Cambridge, Brian Josephson.

We all came to know of Josephson when his name began to appear in the list put out by the mathematician Besicovitch. The list was of those students who solved the problems announced from time to time on the Faculty Notice Board by Besicovitch. So we thought that Josephson would become a pure mathematician.

However, in his final year as an undergraduate, he wrote a research paper pointing out a serious error in an experiment claiming to have proved a prediction of Einstein's general theory of relativity.

As a result of Josephson's paper, the experiment had to be repeated with greater controls to make the claim stick. But that was in physics – at the beginning of Josephson's transition from a mathematician to a physicist. And today all physicists working in low temperature physics know of him for his discovery of the so-called ‘Josephson Junctions’ – the discovery that brought him a Nobel Prize.

These examples describe, in a better way than I could have, what scientific research means to me. I now illustrate my views through personal episodes.

### Seeding

One of my early childhood memories goes back to when I was in Std III. Our class teacher asked all the students: What does your father do? As the school was in the campus of Banaras Hindu University (B.H.U.), most of us were children of university staff members. I recall replying that my father was a professor. “Professor of what?” the teacher asked. I did not know. So the teacher told me: “Your father is a professor of mathematics”. My feeling of inadequacy at not knowing the full answer was instantly replaced by one of elation. I was pleased that my father taught the same subject that I liked best.

I narrate this incident to underscore the fact that my early liking for mathematics was not dictated by my father or by others telling me that I should grow up to be a mathematician just like my father. I know of cases where children are consciously or unconsciously pressurised to emulate the achievements of their parents. It was by observing my father at work that I became enamoured of the life of a researcher. I saw him sitting on the floor with papers spread all around him, trying out long mathematical sums that I knew nothing about. Unlike the sums I did at school, which were short but had definite answer, in his case, as I gathered from my naïve questions, there was *no known answer and* he was trying to find what the answer should be.

That I liked maths and science was noticed by my father, who made me acquainted with the recreational aspects of mathematics, with its wealth of anecdotes, puzzles and paradoxes. He did this either directly or by giving me books of this nature. He also encouraged my brother and me to do experiments. Our house in the B.H.U. campus was spacious enough for him to provide a chemistry lab for my brother and myself to play with.

In those days it was customary for visiting faculty from other universities to stay with their local host and so we had mathematicians like N.R. Sen, Ram Behari, A.C. Banerjee or Vaidyanathaswamy staying with us on such visits. Even if I did not understand what they were talking about, the overall ambience did help in creating an aura about mathematics.

I had a taste of creative thinking myself in standard X. I had managed to find a shorter proof of the famous Pythagoras theorem about the right-angled triangle. It was certainly timesaving and I presented it in the terminal examination that year. My teacher gave me full marks. While patting my back, he also gave me a friendly advice: *Do not give this proof in the Board examination if the question appears there*. For, the examiner may be in a hurry to deal with the large number of scripts he has to evaluate and not seeing the familiar figure, he would give a zero, without bothering to read the proof. I was a little disappointed: but this message also contained a warning that being original or nonconformist in research can bring its own difficulties. I will return to this idea later.

### Growing

However, a crucial development, which helped foster a competitive spirit in me, took place when I was in the VIII standard. My maternal uncle Moreshwar Huzurbazar or *Morumama* as I used to address him, came to live with us in order to study for a M.Sc. in mathematics at the B.H.U. He was a brilliant scholar, having done very well at the B.Sc. exam of Bombay University. [Later in his life he was a professor and then director of the Institute of Science, Bombay.]

*Morumama* discovered that I enjoyed doing mathematics. He also noticed that my father had two blackboards built into the walls for my brother and myself to write or draw as we wished. He found a new use for one of the boards. Once in a while he would write a mathematical problem or puzzle, under the title “Challenge Problem for JVN”. The problem would remain on the board till either I solved it or gave in and asked for the answer (which, I am glad to say, happened rather rarely).

*Morumama's* problems were certainly outside my school syllabus: they called for analytical reasoning and ‘trick solutions’, which would light up for me some hidden aspect of mathematics. My lasting regret has been that no record has been kept of those problems. But so far as I was concerned, I developed an attitude of taking on the challenge posed by a difficult question.

I should mention too that some teachers I encountered at school were also inspiring. Occasionally I would take *Morumama's* problems to school. Mr Pande, my maths teacher, would have time for discussing it, even though he himself could not solve it. How many teachers, overburdened as they are with a large student population and an overstuffed syllabus, can today find time for such excursions into the byways of mathematics? I recall Pandeji taking up a whole period discussing the proof of the so-called *difficult converse*: “If the angle bisectors of the base angles of a triangle are equal, then the triangle is isosceles”.

Perhaps I should mention that books like *‘Men of Mathematics’*, *‘The World of Mathematics’*, *‘Living Biographies of Great Scientists’*, etc, played a key role in bringing to my impressionable mind the excitement and frustrations of creative geniuses. Science is not a drab subject to be memorized, but an arena of adventures. It was revealing to know about the pride and prejudices of great scientists and to learn that they too occasionally made mistakes. But science has a self-correcting mode that leads ultimately to the right answer. This was one motivating influence in my opting for a career in science.

### Decision Making

I have stressed my liking for mathematics, but I should add that I liked physics too. Here, however, my school syllabus was not very exciting and, apart from an occasional puzzle, I did not get to share the excitement of learning and experiencing how nature's laws work. Thus physics was my second favourite and close on its heels came Sanskrit.

So far as my liking for Sanskrit goes, I owe a lot to my late mother and to *Morumama*. My mother inducted me into Kalidasa and Bhavabhuti: identifying the power and beauty of the language, which one can appreciate only through the works of literary geniuses like these. And *Morumama* inducted me into the literary gymnastics and puzzles that this language seems uniquely fit to describe. I wish our university system were flexible enough to allow a science student to do a course in Sanskrit too. But alas, no! After my matriculation I had to make the choice: and I could have Sanskrit only if I opted for arts, giving up science.

But the point of decision-making came at the end of the intermediate science examination, the stage now identified with the higher secondary or standard XII. The B.H.U. had an engineering college with a national reputation (now part of the Institute of Technology). It was difficult to get into and much sought after. I was expected to do well at the I.Sc. Examination and one of the options before me was to go for the engineering degree.

I recall visiting the B.H.U. Engineering College at the time of the annual exhibition put up by students for the general public. In fact I used to visit the exhibition every year and enjoy the clever way machines were used to do work in the models displayed. On this particular occasion, some college faculty members greeted me and said that they hoped to see me as a student there next year.

However, for me the decision was already made. I had developed sufficient attachment to mathematical sciences so that the alternative of opting for engineering did not even enter my mind. The thrill of solving problems whose solutions one did not know must be even greater, I felt, than solving *Morumama's* problems whose solutions were known, at least to him. Such were the problems I had seen my father spend hours working on, with pages of long calculations covering the floor around him.

Indeed my future projections at this stage took me to the Mathematical Tripos at Cambridge, where I felt, one's mettle is really tested. I had decided to try for it after completing my B.Sc. at the B.H.U. My father, who had had a very successful career at Cambridge, was all for it.

When, before going to Cambridge, I called on Mr R.P. Paranjpye, Senior Wrangler at Cambridge of the 1899 vintage, he asked me: “After doing the Mathematical Tripos, will you go for the IAS?” He was voicing a view common in those days, that a Cambridge degree was a good stepping-stone for the Indian Administrative Service. When the great RPP distinguished himself at Cambridge he was expected to join the Indian Civil Service. But he opted for a teaching career.

My answer to Mr Paranjpye was likewise quite definitive: “No Sir, I wish to enter a career of teaching and research”.

### Trials and Tribulations of Non-Conformism

After my Cambridge Tripos I enrolled as a research student of the celebrated astrophysicist Fred Hoyle. In my first briefing session with him, he suggested several problems in astrophysics and cosmology, one of which I could take up for my Ph.D. I found that his own theory, the steady state theory, was missing from his list. I asked him, whether I could work on that theory. He replied that the theory was controversial and as he did not believe in involving a new research student in a controversial topic, he had not included it in his list.

As it happened, within 7-8 months I was willy-nilly drawn into a controversy as the Cambridge radio astronomers, under the leadership of Martin Ryle, came up with results that claimed to have disproved the steady state theory. Hoyle was called upon to react to the claim and, in his rejoinder, he wanted to demonstrate that the steady state theory was in fact consistent with the Cambridge data, given all the realistic uncertainties of observations. In late January Hoyle asked me to work with him on a mathematical model that would back his hunch. Time was short as Ryle was due to present his results to the Royal Astronomical Society in early February, within two weeks or so. I recall working out a model that fitted the data as well as being consistent with the steady state theory.

However, now came the crunch! On the day of the meeting, Hoyle had another unavoidable commitment and so he asked me to present the results. I was nervous at first at the prospect of facing a seasoned adversary like Ryle in a debate. However, Hoyle said that if I was convinced our work was correct, I could argue for it against any adversary. So I did, on that second Friday of February 1961. I could carry my side of the argument well and several neutral people in the audience came to tell me that they could understand and appreciate my reasoning.

From that day onwards I found a new confidence in research. Even if its findings are against the beliefs of the majority, if one's research is consistent with facts and has been correctly worked out, then it can and should, be defended with confidence.

I have carried that message with me throughout my 4-5 decades of research. I have often worked on unconventional ideas. One realizes that one is batting on an uneven pitch against many hostile factors. There are many frustrations, like unkind remarks of a referee examining your paper, the reluctance of conference organizers to allow time for expressions of anti-Establishment views etc. By one's identification as a maverick one may occasionally miss an honour or an award. One may not be elected Fellow of a Learned Society. But the excitement of saying something new ultimately carries the day. As Fred Hoyle used to say: “If the conventional path were correct, then with so many bright brains working on it they would have reached success by now. The fact that this has not happened, lends hope to those who dare deviate from the path chosen by the Establishment”.

### Moments of Creativity

So this is what scientific research means to me: the fun and excitement of walking on uncharted territory and finding golden nuggets in unexpected places, because one dared deviate from the recommended path.

Perhaps I may mention one of those moments when you suddenly find what you have been looking for and having found it, you are both happy at the discovery and a little chagrined that you did not think of it before. I was looking for a way to describe how physical interaction between two distant particles can be described in a space-time structure that does not obey Euclid's simple geometry. I had spent several weeks trying out different approaches until one day I came across a paper by two US scientists DeWitt and Brehme who had looked at a different question and had used a new technique to solve it. I could immediately see that this technique was what I was looking for, if adapted to my requirements. From then on, everything fell into its place and I felt that the road to success was suddenly opened. This was a “Eureka” moment for me.

Working with Fred Hoyle was itself a revelation. He had intuition in plenty and it was uninhibited because he never felt constrained by ‘standard’ ideas. Which is why he could spot a solution that had eluded others. For a long time scientists were trying to see how nuclear fusion in stars could progress beyond the nucleus of helium. If you try to synthesize a bigger nucleus by combining hydrogen with helium or by bringing two helium nuclei together, you discover that the resulting nucleus is unstable and it breaks back. People had thought of combining three helium nuclei to make carbon, that is a stable nucleus. But that was a very rare event: bringing three helium nuclei together was a difficult thing to achieve by random encounter. Hoyle had a brain wave. If the event is rare, one needs a reaction to go fast to compensate the rarity-effect. And the reaction would run fast if it had resonance; that is, if the energy of the participating helium nuclei exactly matched the energy of carbon formed. Hoyle estimated that this would happen if the carbon nucleus were in an excited state. So he asked nuclear physicists at Caltech to check if an excited state of carbon with that energy did exist. At first they did not believe him, but at his persistence went ahead and did find such an excited state. Why was Hoyle so persistent? Because his intuition told him that with so much carbon in the universe, there had got to be some way of making it and the stars provided the only setting!

What leads to greater creativity? Is it to do with personal lifestyle? Does a tranquil lifestyle help? Or should one be always working under stress? My own experience has been for the former. A relaxed environment, where one can think without external issues to bother one, is the ideal scenario. However, it is not sufficient! Unless one has ideas to think about, one cannot hope to be creative and to be creative one must be totally immersed in the subject.

### Take Home Message

For doing good research, one must genuinely like the subject one is working on. Indeed a professional research worker should be able to say that he is fortunate in having his hobby for his profession.

## Questions That This Paper Raises

1. Give an example from your own field of interest to illustrate the difference between a research problem and an examination problem.
2. Look up from the web the story of the brachistochrone problem. Have you ever been driven to work on a problem like Newton was in this case?
3. It is said that Newton thought of the inverse square law of gravitation when an apple from a tree fell upon him. To what extent could this legend be true?
4. Suppose you have to colour a map on the globe in which different countries sharing a common border are painted in different colours. What is the least number of colours needed for this exercise? After trying to solve this problem, look up the history of the “Four colour Problem” on the website.

## About the Author

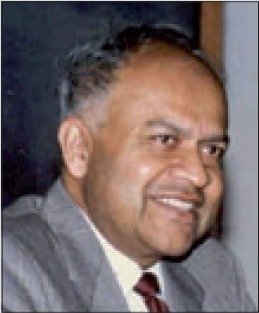



Jayant Vishnu Narlikar was born on July 19, 1938 in Kolhapur, India. He was educated in the campus of the Banaras Hindu University and later in the University of Cambridge. He did a Ph.D. degree after completing his Mathematical Tripos examinations and becoming a Wrangler. He stayed on in Cambridge for a brief post-doctoral career as Fellow, King's College (1963-72) and Staff Member of Institute of Theoretical Astronomy (1966-72). He returned to India in 1972 to join the Tata Institute of Fundamental Research in Mumbai as Professor and later in 1989 moved to Pune to set up the Inter-University Centre for Astronomy and Astrophysics (IUCAA) of which he was the Founder Director till 2003. He has worked in gravitation and astrophysics and was the President of the Cosmology Commission of the International Astronomical Union for 1994-97. He has also distinguished himself as a science populariser. He is married to Dr Mangala Narlikar, a mathematician and has three daughters Geeta, Girija and Leelavati, all doing research in different branches of science.

